# Stony Brook University Forging Intergenerational Links Through Life-Story Sharing Project

**DOI:** 10.1093/geroni/igaa057.1745

**Published:** 2020-12-16

**Authors:** Giselle Ferguson, Caitlin Monahan, Sheri Levy, Suparna Rajaram, Lauren Richmond, Stacey Scott

**Affiliations:** Stony Brook University, Stony Brook, New York, United States

## Abstract

According to the World Health Organization, the global population is aging, but ageism may now be the most socially “normalized” of any prejudice, more pervasive than sexism or racism. Ageism produces avoidant and disrespectful treatment of older adults and contributes to a shortage of college students seeking careers with older adults. To foster positive intergenerational contact and combat ageism, we organized life-story sharing by older adult community members in four undergraduate psychology courses with lifespan themes (Psychology of Aging, Memory, Death & Dying, Developmental Psychology; n≅500). A panel visited each class; instructors and graduate students facilitated discussion between students and panelists. Students completed pre- and post-surveys of ageism and attitudes toward aging. A majority of students recommended integrating the activity into future semesters. In free-responses, students also frequently expressed surprise that panelists reported not feeling different than they had at age 20, and that this information challenged previously held stereotypes.

